# Gene expression profiling of reactive oxygen species (ROS) and antioxidant defense system following *Sugarcane mosaic virus* (SCMV) infection

**DOI:** 10.1186/s12870-020-02737-1

**Published:** 2020-11-23

**Authors:** Sehrish Akbar, Yao Wei, Yuan Yuan, Muhammad Tahir Khan, Lifang Qin, Charles A. Powell, Baoshan Chen, Muqing Zhang

**Affiliations:** 1 State Key Laboratory for Conservation and Utilization of Agro Bioresources, Guangxi Key Laboratory for Sugarcane Biology, Guangxi University, Nanning, 530005 China; 2 Nuclear Institute of Agriculture (NIA), Tando Jam, 70060 Pakistan; 3 IRREC-IFAS, University of Florida, Fort Pierce, FL 34945 USA

**Keywords:** *Sugarcane mosaic virus*, Reactive oxygen species, Transcriptome, Antioxidant, Transgenic

## Abstract

**Background:**

Viruses are infectious pathogens, and plant virus epidemics can have devastating consequences to crop yield and quality. *Sugarcane mosaic virus* (SCMV, belonging to family *Potyviridae)* is one of the leading pathogens that affect the sugarcane crop every year. To combat the pathogens’ attack, plants generate reactive oxygen species (ROS) as the first line of defense whose sophisticated balance is achieved through well-organized antioxidant scavenging pathways.

**Results:**

In this study, we investigated the changes occurring at the transcriptomic level of ROS associated and ROS detoxification pathways of SCMV resistant (B-48) and susceptible (Badila) sugarcane genotypes, using *Saccharum spontaneum* L. genome assembly as a reference genome. Transcriptomic data highlighted the significant upregulation of ROS producing genes such as NADH oxidase, malate dehydrogenase and flavin-binding monooxygenase, in Badila genotype after SCMV pathogenicity. To scavenge the ROS, the Badila genotype illustrated a substantial enhancement of antioxidants i.e. glutathione s-transferase (GST), as compared to its resistant counterpart. GST is supposed to be a key indicator of pathogen attacks on the plant. A remarkably lower GST expression in B-48, as compared to Badila, indicated the development of resistance in this genotype. Additionally, we characterized the critical transcription factors (TFs) involved in endowing resistance to B-48. Among these, WRKY, AP2, NAC, bZIP, and bHLH showed enhanced expression in the B-48 genotype. Our results also confirmed the linkage of transcriptomic data with the enzymatic and qPCR data. The estimation of enzymatic activities for superoxide dismutase, catalase, ascorbate peroxidase, and phenylalanine ammonia-lyase supported the transcriptomic data and evinced higher resistance in B-48 genotype.

**Conclusion:**

The current study supported the efficiency of the B-48 genotype under SCMV infection. Moreover, comparative transcriptomic data has been presented to highlight the role of significant transcription factors conferring resistance to this genotype. This study provides an in-depth knowledge of the expression profiling of defense mechanisms in sugarcane.

**Supplementary Information:**

The online version contains supplementary material available at 10.1186/s12870-020-02737-1.

## Background

Naturally, plants are under continuous threat of infection by numerous pathogens such as bacteria, viruses, and fungi. Among these, viruses are obligate intracellular parasites, which cause enormous loss to crop yield and quality every year. *Sugarcane mosaic virus* (SCMV), *Sorghum mosaic virus* (SrMV), and *Sugarcane streak mosaic virus* (SCSMV) are the leading pathogens of sugarcane. These viruses cause mosaic disease of the sugarcane crop resulting in significant yield losses. In China, sugarcane ratoon stunting, pokkah boeng, smut, mosaic, and other diseases are responsible for up to 20% reduction in sugarcane production [1].

Plants have evolved multiple strategies to circumvent the pathogenic infection. The first line of defense against these obligate intracellular parasites is the rapid generation of reactive oxygen species (ROS), e.g.*,* hydrogen peroxide (H_2_O_2_) and superoxide anions (O^− 2^) [2] (Additional file 1: Figure S1). The chloroplast is the primary organelle where ROS are produced during the photosynthetic electron transport chain (ETC) [3, 4]. Besides, they are also produced in mitochondria during aerobic respiration and in peroxisomes during photorespiration [5–7]. ROS act as signaling molecules which play a vital role in plant growth and defense mechanism as they are involved in instigating the production of the antioxidant in plant cells [8, 9]. In infected plants, the amassing ROS has a dual role to play. They may elevate the programmed cell death (PCD) of pathogenic cells, and confinement of invading pathogens to the infected parts. However, at high concentration, ROS are incredibly detrimental, and may ultimately result in disruption of cellular homeostasis. Elevated ROS production can cause cellular damage through peroxidation of lipids, protein oxidation, nucleic acid degradation, enzyme inhibition, and initiation of PCD pathways that eventually induce cell death [10–13].

Scavenging or detoxification of the surfeit ROS concentrations is carried out through a well-organized antioxidative system, constituted by the non-enzymatic as well as enzymatic antioxidants. The enzymatic antioxidants encompass catalase (CAT, EC 1.11.1.6), superoxide dismutase (SOD, EC 1.15.1.1), guaiacol peroxidase (GPX, EC 1. 11.1.9), dehydroascorbate reductase (DHAR, EC 1.8.5.1), monodehydroascorbate reductase (MDAR, EC 1.6.5.4), ascorbate peroxidase (APX, EC 1.11.1.11), and glutathione reductase (GR, EC 1.8.1.7) [14]. While, ascorbate, glutathione (GSH), tocopherols, phenolics, and carotenoids are considered as potent non-enzymatic antioxidants within the cell. In an antioxidant defense mechanism, SOD catalyzes the conversion of oxygen radicals to H_2_O_2_, which is later reduced to water and molecular oxygen through biochemical reactions catalyzed by CAT, POD, and APX enzymes. These conversions by antioxidant defense systems protect the cell membrane from injury by oxygen radicals [15].

Plant positive-strand RNA viruses [(+) RNA viruses] are obligate parasites. Their multiplication relies on protein factors and metabolites of the host [16, 17]. Although ROS bursts are associated with the effective plant virus infection and initiation of the disease, the mechanisms through which ROS influence the viral infection have not been extensively apprehended. It has been established that positive-sense RNA viruses (+sRNA) depend on host protein machinery for replication [18]. Subsequently, ROS burst is initiated in plants, once the virus hijacks their cellular machinery. Hyodo et al. [16] investigated *Red clover necrotic mosaic virus* (RCNMV) infection and proposed that the ROS generating enzyme of *Nicotiana benthamiana* is triggered by the RCNMV replication protein, which facilitates the virus replication and induces intracellular ROS burst. Thus far, only limited data is available regarding the correlation between pathogenicity level and ROS production in sugarcane following the SCMV infection. Hence, there is a dire need to explore the status of ROS engenderment and the extent of damage to plant cells after SCMV infection.

Keeping in view the limitations of sugarcane genomics and disease resistance mechanisms data, we extended our study to provide insights into the antioxidant defense system in SCMV resistant genotype. Previously, we have developed transgenic sugarcane lines through particle bombardment of coat protein (CP) of SCMV strain E, named as B-48. Transgenic B-48 lines were highly resistant against SCMV infection, with higher photosynthetic activity [19]. In the current study, we compared wild type (Badila) and transgenic (B-48) genotypes to understand the regulation of defense response at the transcriptional and enzymatic level. The objective of our study was to perform comparative analysis for evaluating the ROS production and antioxidant defense system. This study will provide valuable information regarding SCMV pathogenicity and defense associated pathways in sugarcane.

## Results

### ROS production of sugarcane infected by SCMV virus

To determine the ROS production, H_2_O_2_ contents were estimated in Badila and B-48 genotypes. For evaluating the B-48 transgene resistance level against the SCMV pathogenicity, we estimated H_2_O_2_ production in both the genotypes before-inoculation (BI) as well as post-inoculation (PI).

**Fig. 1:**
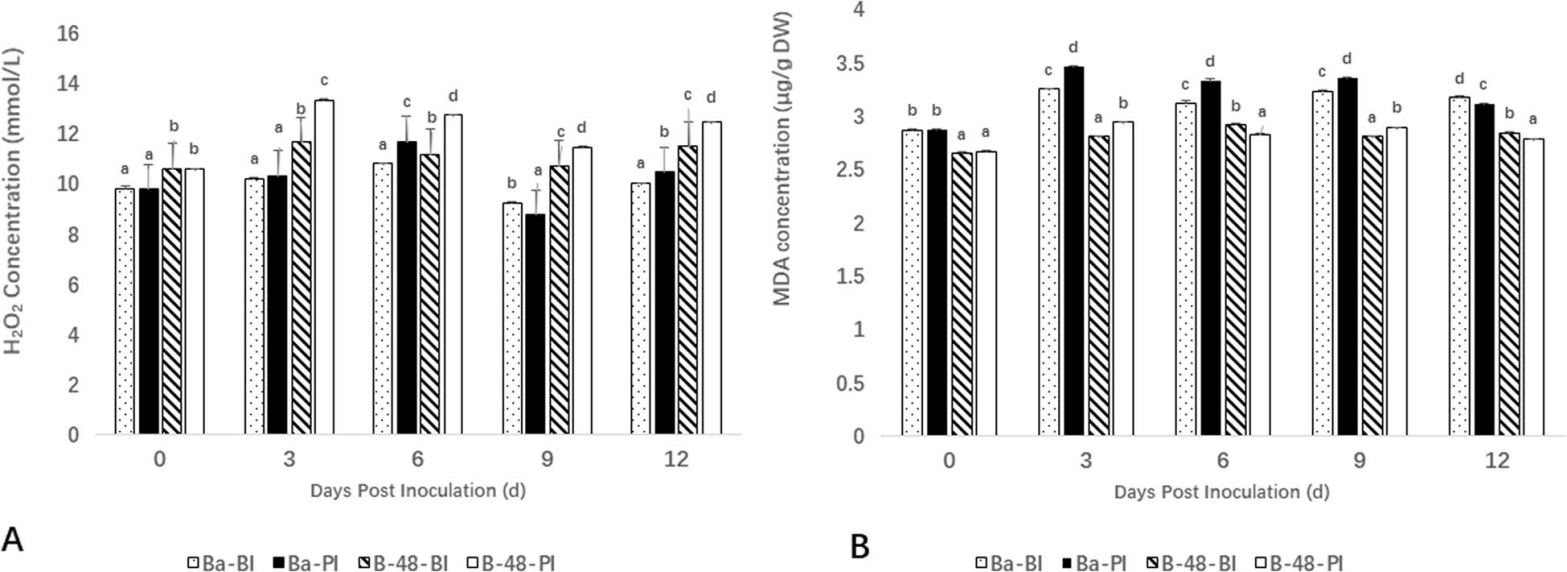
Determination of hydrogen peroxide (H_2_O_2_) contents (A) and Melonaldehyde (MDA) contents (B) in Badila and B-48 genotypes before and after virus infection (*P* < 0.05)

It was observed that initially, there was a sharp increase in H_2_O_2_ contents in the B-48 genotype as compared to Badila, till 3rd day. However, the level of H_2_O_2_ increased slightly in Badila genotype at 6th day. At 9th day, there is maximum decline in H_2_O_2_ content in both genotypes. Further, at the 12th day there is a little increase in H_2_O_2_ content in both genotypes, but lower as compared to the 3rd and 6th day (Fig. 1A).

### Lipid peroxidation in response to SCMV infection

The lipid peroxidation level was determined by estimating the malondialdehyde (MDA) contents. Noticeably, the MDA level was higher in post-inoculated Badila (PI) vs. the before-inoculated Badila, B-48 (BI), and B-48 (PI) samples. The SCMV infection caused maximum cellular damage in Badila (PI) sample by day 3, which was evident from its MDA levels. The MDA contents of the transgenic line were relatively low, specifically at 6th, 9th and 12th days of measurements indicating less lipid peroxidation, attributed to the genotypic tolerance against SCMV induced stress (Fig. 1B).

### Gene expression profiling of ROS producing and ROS scavenging pathways

Due to the unavailability of the *Saccharum officinarum* L. genome sequence, we used the *Saccharum spontaneum* L. database as a reference genome [20]. However, the major limitation in this reference was the reduction in the genome size from ten basic chromosome numbers to eight in the *Saccharum spontaneum* genome. Despite the genome reduction, the overall reference sequence database represented a 69–74% alignment rate (Additional file 2: Table S1). We used the Kyoto Encyclopedia of Genes and Genomes (KEGG) web database for the annotation of ROS and antioxidant genes. KEGG automatic annotation server (KAAS) and KEGG mapper online tools were used to annotate and map the KEGG database descriptions. In total, we identified 757 transcripts related to ROS and antioxidant defense associated pathways in Badila and B-48 genotypes (Additional file 3: Table S2).

Under stress conditions, plant cells scavenge excessive ROS perturbation by various antioxidative defense systems. The antioxidant defense mechanisms are classified into two types viz. enzymatic and non-enzymatic systems. In the current study, we divided the identified transcripts into ROS producing and ROS scavenging categories. Among the total 757 transcripts, 329 were associated directly or indirectly to ROS production, while 428 were involved in ROS scavenging pathways. The antioxidant defense pathway was further divided into two groups, which contained 399 transcripts involved in the non-enzymatic pathway and 29 transcripts associated with the enzymatic pathway.

### Comparative transcriptional profiling of ROS genes in Badila and B-48 before and post SCMV infection

**Fig. 2:**
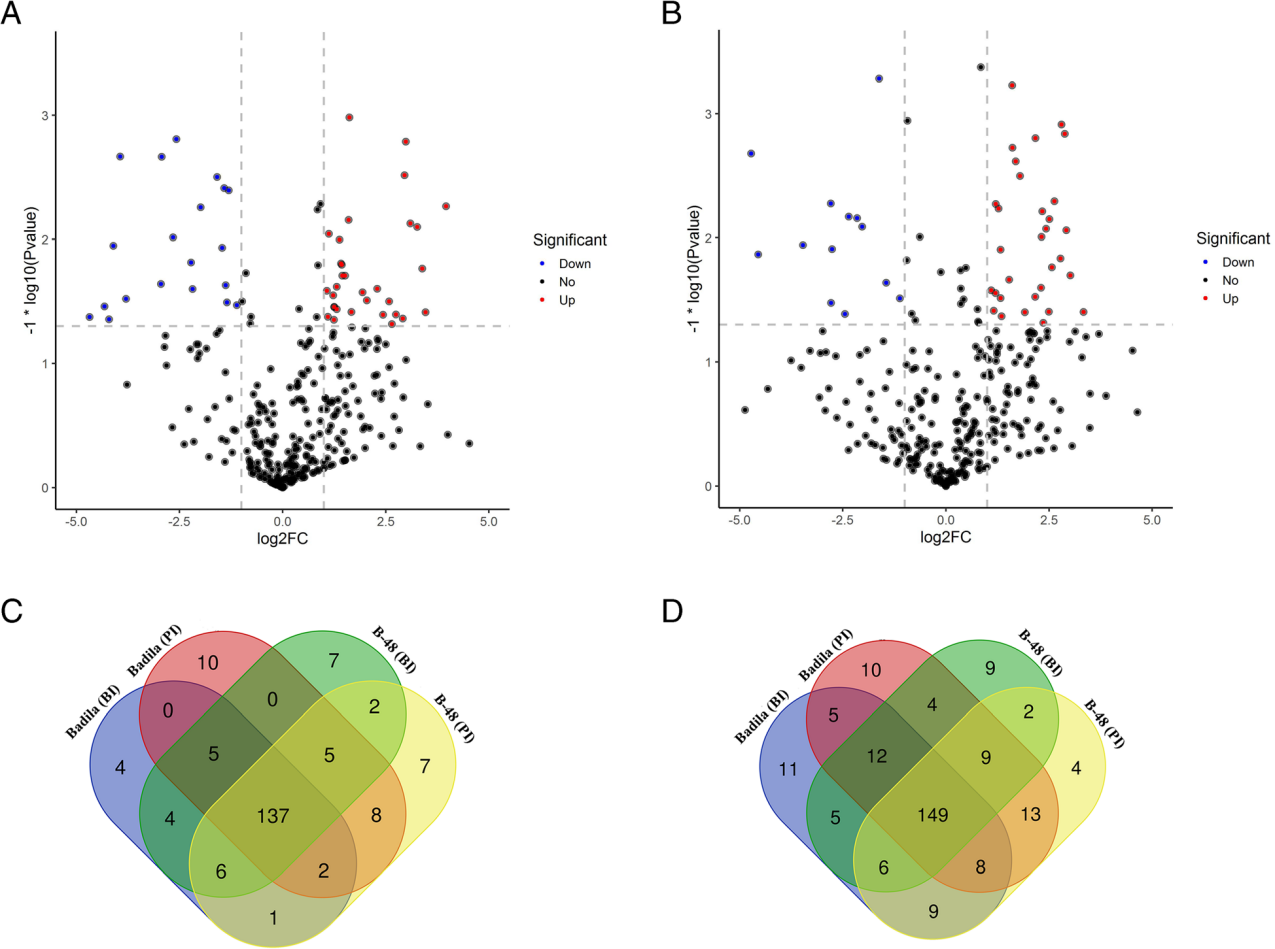
Number of transcripts (**a**, **b**) and expression pattern (**c**, **d**) of ROS-associated genes or transcripts in Badila (Ba) and B-48 before and post SCMV infection. **a:** Number of overlapped and unique ROS transcripts; **b:** Number of overlapped and unique antioxidant transcripts; **c**: Expression pattern of ROS genes; **d**: Expression of antioxidant genes. The differential expression was considered as significant at log_2_FC, *p* < 0.05. The differential expression analysis showed 32 upregulated and 25 downregulated genes

To understand the mechanism of SCMV infection in Badila and B-48 genotypes, we compared the transcriptional profiles before and after the SCMV infection. The differential expression was considered significant at False Discovery rate (FDR) < 0.05 and Fold Change > 2. The differential gene expression analysis showed that the SCMV pathogenicity perturbed a total of 329 differentially expressed genes (DEGs) associated with the ROS production. Among the identified DEGs, 32 were upregulated, 25 were down-regulated, while 272 did not show significant differential response in Badila and B-48 genotypes, post-SCMV infection (Fig. 2a).

### Comparative transcriptional profiling of antioxidant genes in Badila and B-48 (before and post SCMV infection)

We performed the comparative transcriptional profiling of antioxidant genes in Badila and B-48 genotypes, before and post SCMV infection. This analysis facilitated the determination of the number of DEGs at FDR < 0.05 and Fold change > 2. The SCMV infection caused differential expression of 197 antioxidants-related DEGs in Badila and B-48, of which only five DEGs were upregulated, while 20 were down-regulated. The remaining 172 did not represent a significant differential response (Fig. 2b).

The number of unique and shared transcripts associated with ROS and antioxidant pathways were identified in Badila and B-48 before and post SCMV infection. Interestingly, 137 ROS-generating (Fig. 2c) and 149 ROS-scavenging (Fig. 2d) transcripts were shared by Badila and B-48 genotypes. However, 14 and 16 unigenes were unique in Badila and B-48 genotypes, respectively, in ROS generating pathway. While 26 and 15 unigenes were unique in Badila and B-48 genotypes, respectively, in the ROS scavenging pathway.

### Differential gene expression analysis of ROS producing transcripts in response to SCMV infection

**Fig. 3:**
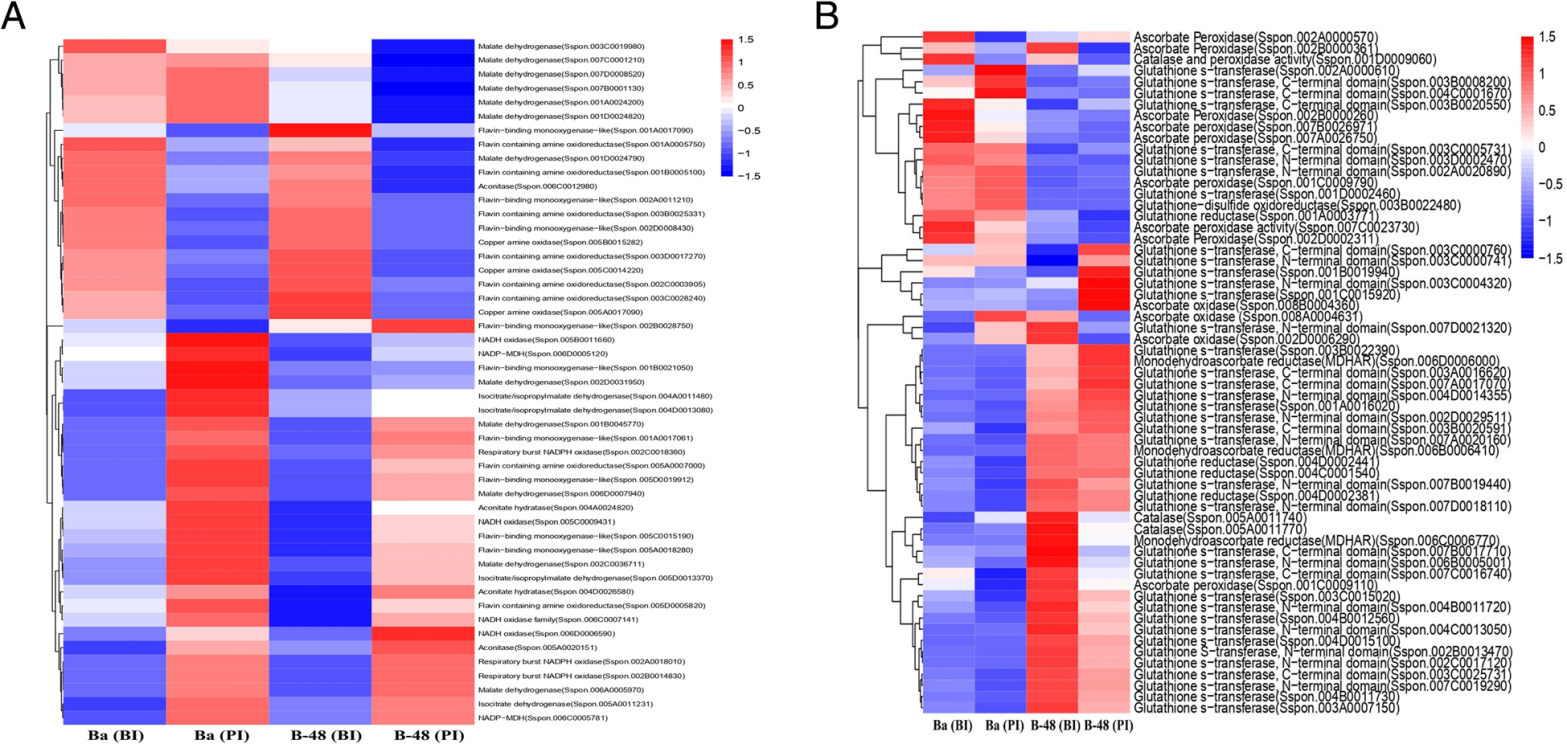
Number of differentially expressed genes involved in ROS producing (**a**) and scavenging (**b**) pathway in Badila (Ba) and B-48 before and post SCMV infection. The heatmap, developed using heatmap package, represented the value of the transcripts expression level fold change (log_2_FC)

To evaluate the effect of SCMV infection, we performed comparative gene expression profiling between Badila and B-48 genotypes and distinguished the ROS producing DEGs. Approximately 49 highly differentiating DEGs were selected for gene expression analysis (Fig. 3a). Following exposure of the virus, Badila and B-48 upregulated different ROS-producing genes. The Badila genotype upregulated a total of 28 unigenes while B-48 upregulated only 22. Post-inoculation (PI) Badila showed enhanced expression of four unigenes of NADPH oxidase, four of flavin binding monooxygenase, five of malate dehydrogenase, two of NADP-MDH, four of isocitrate, two of flavin-containing amine oxidoreductase, two of aconitate hydratase, and one of aconitase unigene. Similarly, post-inoculation B-48 (PI) exhibited augmented expression of three unigenes of NADPH oxidase, five of flavin binding monooxygenase, four of malate dehydrogenase, one of NADP-MDH, two of isocitrate, two of flavin-containing amine oxidoreductase, one of aconitate hydratase, and one of aconitase unigene.

### DEGs responsible for ROS scavenging pathway in response to SCMV infection

To examine the effect of SCMV infection on the ROS scavenging pathway, DEGs associated with antioxidants were systematically analyzed. About 61 DEGs related to antioxidant defense pathways were studied for gene expression analysis (Fig. 3b).

Badila maximized the expression of 38 DEGs under the SCMV stress. Among them, twenty-five DEGs were associated with GST, three with glutathione reductase, three with monodehydroascorbate reductase (MDHAR), two (each) with ascorbate peroxidase, ascorbate oxidase and catalase, and one unigene linked with catalase and peroxidase activity. Contrarily, B-48 exhibited a rise in a lesser number of DEGs associated with antioxidants. The upregulated unigenes encompassed about twenty-six for GST, three for glutathione reductase, two for monodehydroascorbate reductase (MDHAR), and one each for ascorbate oxidase and peroxidase.

### Expression profiling of transcription factors (TFs) involved in plant-pathogen defense mechanism

**Fig. 4:**
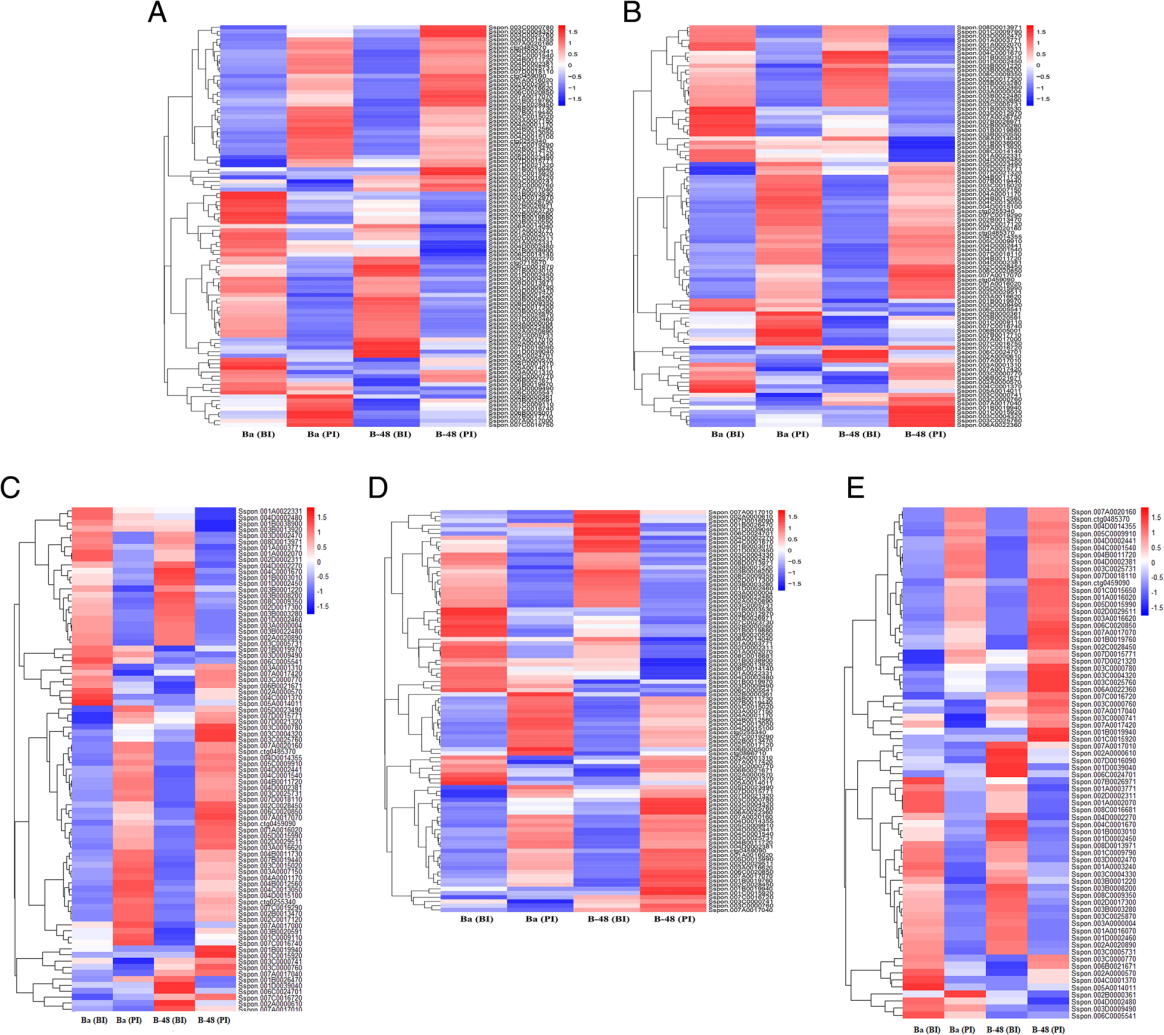
Differential gene expression analysis of WRKY (**a**), AP2 (**b**), NAC (**c**), pZIP (**d**) and bHLH (**e**) transcription factor associated genes in Badila (Ba) and B-48 before and post SCMV infection

Transcriptome profiling was performed to identify the transcription factors involved in defense response against the SCMV infection. We identified five different types of transcription factors that are mainly associated with antiviral defense mechanisms, such as AP2, WRKY, HLH, NAC, and ZIP. The transcriptome analysis revealed that many WRKY genes were expressed following the SCMV infection (Fig. 4a). A total of 304 WRKY genes were identified; among them, we randomly selected 52 DEGs for gene expression analysis. It was noticed that after SCMV infection, B-48 upregulated about 40 DEGs as compared to 32 upregulated DEGs in Badila.

We also analyzed AP2 transcription factors, which make one of the largest families of TFs. AP2 TF is known to be involved in defense mechanisms against numerous plant pathogens. To understand the correlation between AP2 gene expression and disease resistance, we performed the expression profiling of selected DEGs. A total of 404 AP2 DEGs were identified from the transcriptomic data (Fig. 4b). Among the identified DEGs related to AP2, we selected 85 DEGs for heatmap analysis. Overall, the higher number of AP2 DEGs were enhanced in the B-48 genotype after exposure to SCMV, as compared to B-48 (BI), Badila (BI), and Badila (PI) genotypes.

Another family of transcription factors that perform diverse functions during biotic and abiotic stress conditions is the NAC family. In response to SCMV infection, we found a total of 88 NAC transcripts (Fig. 4c). Among these, B-48 upregulated 50 NAC DEGs post virus infection, demonstrating the upsurge of the highest number of NAC DEGs to develop resistance against SCMV infection.

Moreover, the pZIP (Zrt/Irt-like Protein) family of transcription factors was analyzed. Like other dissected TFs, pZIP TFs are also involved in plant defense mechanisms. For pZIP TFs, we identified a total of 323 DEGs. Among them, 83 DEGs were differentially expressed among the genotypes. Parallel to the expression pattern of other TFs involved in pathogen resistance, pZIP transcripts also showed maximum expression in B-48, post virus infection (Fig. 4d).

Similarly, we investigated the basic helix-loop-helix (bHLH) family of transcription factors, which is also an important role player in providing defense against pathogens and conferring resistance to the B-48 genotype post-SCMV infection. In total, 119 bHLH TFs related DEGs were found. Among these, only 72 highly expressed bHLH DEGs were analyzed for gene expression profiling (Fig. 4e). We found that the B-48 genotype illustrated greater expression of bHLH DEGs as compared to Badila. After SCMV infection, the bHLH expression level was high in B-48, suggesting an association of bHLH DEGs to the disease resistance mechanism.

## Estimation of antioxidant enzymatic activity

**Fig. 5:**
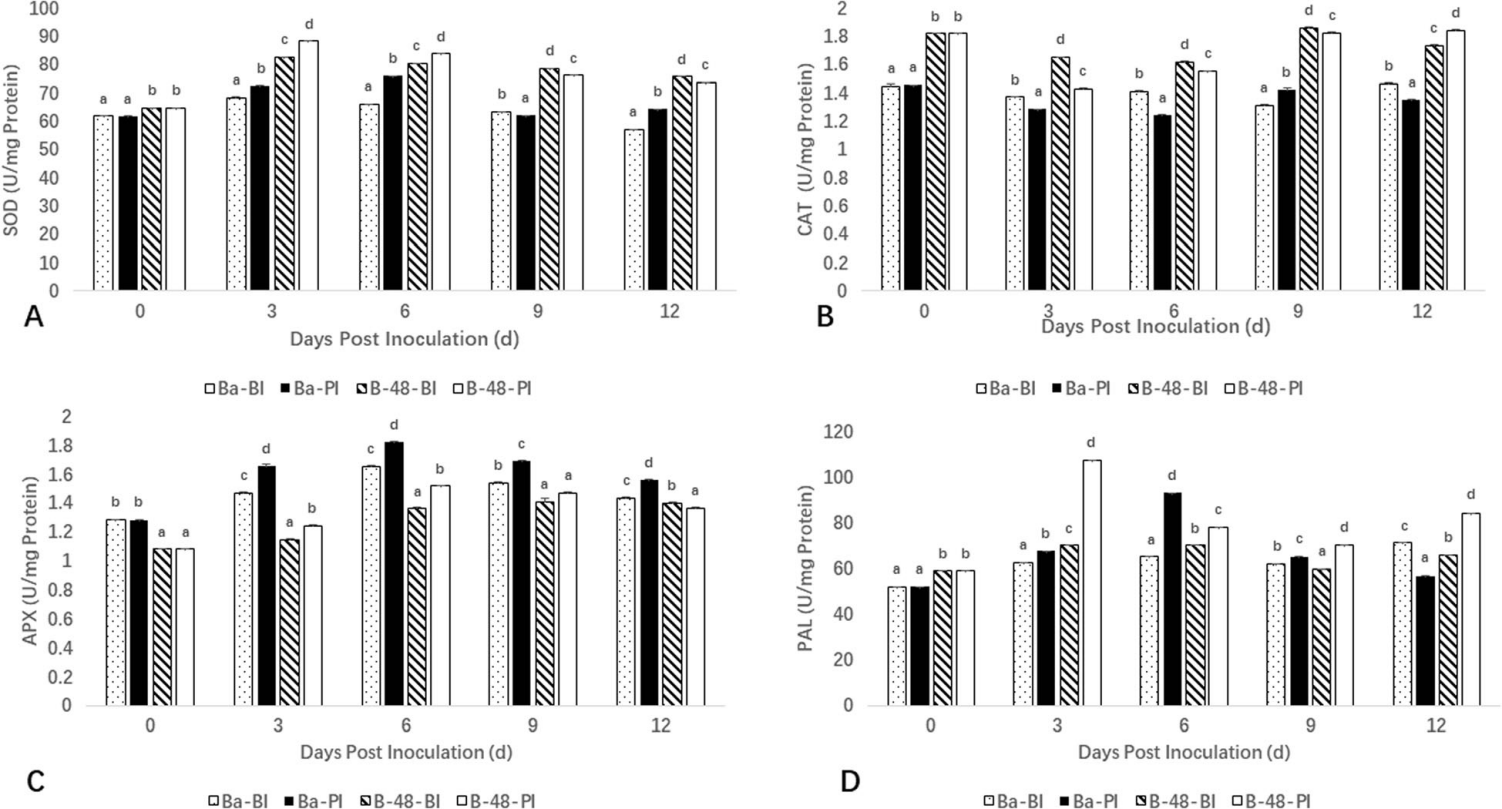
Activities of Superoxide dismutase (A), Catalase (B), Ascorbate peroxidase (C) and Phenylalanine ammonia-lyase (D) in Badila (Ba) and B-48 before and post SCMV infection (*P* < 0.05)

Estimation of antioxidant activities was conducted for SOD, CAT, APX, and phenylalanine ammonia-lyase (PAL), in order to investigate the genotypes’ potential against SCMV triggered stress. The activity of SOD was substantially influenced by SCMV infection in Badila and B-48 genotypes. SOD activity increased till day 3 following viral infection in the B-48 genotype and then continuously decreased at 6th, 9th and 12th days. SOD levels were observed to be higher in B-48 samples as compared to the Badila samples throughout the period of the study. However, maximum SOD expression was visible at 6th day in Badila genotype (Fig. 5A).

The measurement of CAT production levels depicted that SCMV pathogenicity significantly affected the CAT activity. CAT activity was the lowest in Badila (PI) on the 3rd and 6th day of measurement, and markedly increased on the 9th day. The CAT activity in B-48 was found to be close to the CAT activity in Badila, with almost constant production at 3rd, 6th, 9th and 12th days of measurement (Fig. 5B).

The differential response was monitored for APX activity as well in the Badila and B-48 samples. The B-48 (PI) and Badila (PI) depicted higher response as compared to B-48 (BI) and Badila (BI) samples. The highest expression of APX was measured on the 6th day of infection, after which it underwent a sharp decline at day 9th and 12th, in both genotypes (Fig. 5C).

The analysis of PAL activity revealed that the PAL contents reached their maximum level on the 3rd day of measurement in the B-48 genotype post-SCMV infection. Contrarily, PAL activity showed its highest expression on the 6th day of measurement in the Badila (PI) sample. At 9th day, there is decline in PAL activity in Badila (PI) and B-48 (PI). However, at 12th day PAL activity is still lower in Badila (PI) while there is little bit increase in PAL production in B-48 (PI). We noticed a persistent level of PAL in Badila and B-48 genotypes before the viral infection (Fig. 5D).

## Transcriptomic profile validation through qRT-PCR

**Fig. 6:**
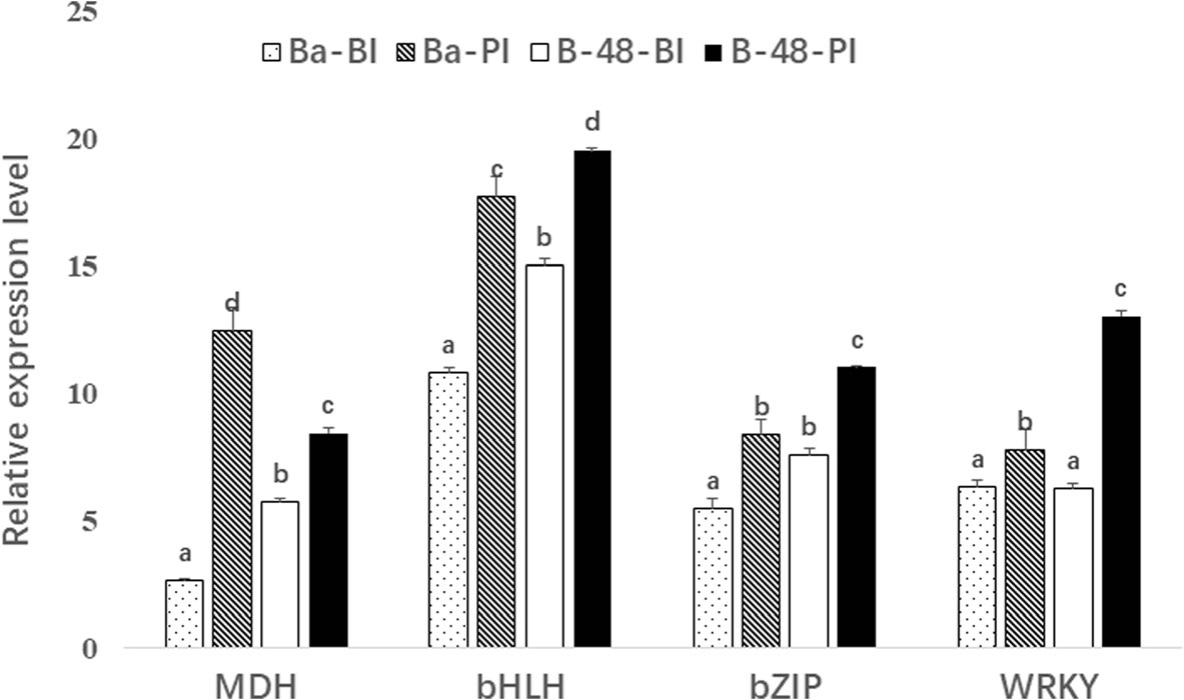
Validation of selected genes in Ba (Before Inoculation) (BI), Ba (Post Inoculation) (PI), B-48 (Before Inoculation) (BI), and B-48 (Post Inoculation) (PI) by Real-Time PCR. The y-axis representing the expression values of the genes, and the x-axis representing the selected genes (MDH: malate dehydrogenase, bHLH: helix loop helix, bZIP: basic leucine zipper and WRKY). Error bars indicated the standard deviation. Data presented in the graph are the combined results of three replicates for all samples. (*p* < 0.05)

To confirm the gene expression data obtained from RNA sequence analysis, we performed qRT-PCR on five selected candidate genes. Primers’ sequences are listed in Additional file 4 (Table S3). qRT-PCR data of the selected genes agreed with the transcriptome data and thus validated the findings of RNA-seq analysis. B-48 showed higher expression of bHLH, bZIP, and WRKY, which are involved in ROS scavenging pathways. Hence, the B-48 genotype was anticipated to be having more resistance against SMCV infection (Fig. 6).

## Discussion

ROS plays a significant role in response to biotic and abiotic stress conditions. Here, we discussed the function of ROS and antioxidant defense mechanisms during pathogen infection, highlighting the interaction of ROS linked transcription factors with the plant pathogen-resistance mechanisms. Furthermore, we have shown that B-48, a transgenic sugarcane cultivar, is resistant to SCMV and demonstrates reduced levels of ROS following the infection of this virus.

The initiation of lipid peroxidation is another indicator of oxidative stress; it begins when the ROS production is above the threshold levels. The lipid peroxidation under biotic and abiotic stresses out-turns in the production of MDA [21]. In this study, we observed that after a sudden rise in H_2_O_2_ and MDA contents, there was a continuous decline in their concentrations in the B-48 genotype. The observation could be attributed to the resistance of B-48 against SCMV, which resulted in alleviation of H_2_O_2_ and MDA levels as compared to the corresponding control. These findings were consistent with the previous report of Radwan et al. [22] who mentioned higher H_2_O_2_ and MDA concentrations in leaves of *Vicia faba* after *bean yellow mosaic virus* infection.

ROS are produced through multiple pathways in several plant tissues, during different developmental stages, and under various conditions. ROS are formed by the activity of a group of enzymes, including cell wall peroxidases, amine oxidases, NADPH oxidases, oxalate oxidases, lipoxygenases, and quinone reductase [23, 24]. The transcriptomic approaches have facilitated to decipher the genetic regulation of ROS production and ROS scavenging pathways in response to SCMV infection. In the current study, the production of ROS was more evident in the Badila genotype after SCMV infection, which showed a higher expression of ROS regulating DEGs insinuating more disruption of the cell ultrastructure and, ultimately, cell death of the infected plants. Comparatively, the B-48 genotype showed resistance to virus infection by upregulating only a limited number of ROS generating transcripts.

This study highlighted the differential expression pattern of the antioxidant genes involved in the defense pathway following SCMV infection. Data showed that the antioxidant defense mechanism was activated in response to the infection, in order to restore the redox homeostasis. We observed the significant expression levels of GST in the Badila genotype, while its expression in B-48 was substantially low. Previous transcriptomic research has supported the marked induction of specific GST groups during the early phase of bacterial, fungal, and viral infection. Likewise, preceding proteomic data has supported the upregulation of GST proteins during pathogen infection. Moreover, it has also been reported that the overexpression and silencing specific groups of GSTs significantly reduce the disease severity and pathogen multiplication [25].

The transcriptomic data were further verified through antioxidative enzymatic assays. In this study, the activity of SOD, which provides defense at initial stages of viral infection, rapidly increased in the B-48 genotype; the same was the case with the PAL enzyme. Moreover, APX activity also plays the most prominent role in the ROS scavenging pathway. Increased expression of APX in Badila and B-48 genotype indicated increasing tolerance against the disease, as reported previously [26, 27]. Additionally, CAT is also an important enzyme involved in detoxification of H_2_O_2_. The CAT contents were higher in transgenic genotype throughout the period of the study. A decline in the CAT activity of both genotypes following virus inoculation was parallel to earlier reports of the plant-pathogen system [27–30].

The transcription factors are DNA binding proteins. Many of them are significant components of plant defense mechanisms. Transcription factors such as WRKY, APETALA2 (AP2), basic domain leucine-zipper (bZIP), NAM/ATAF/CUC (NAC), and bHLH are associated with plant-pathogen disease resistance mechanisms [31–33]. Overall, in this study, we ascertained an increasing trend in the expression of defense-related transcription factors in the B-48 genotype after exposure to the SCMV.

One of the largest family of transcription factors is WRKY, whose individual members play a significant role in the plant defense system. Previously, it has been reported that mutation in the WRKY8 gene caused accumulation and systemic infection of the *Tobacco mosaic virus* (TMV) in *Arabidopsis thaliana* leaves. WRKY8 gene enhances the antiviral defense system through upregulating the abscisic acid (ABA) signaling pathway while downregulating the ethylene signaling pathways [34]. Another family of TFs involved in the defense pathway is AP2. Previous studies have revealed that the AP2 activates the defense-related genes, for instance, PLANT DEFENSIN 1.1/1.2 (PDF 1.1/1.2) [35].

The expression of the NAC family of TFs is also modulated in plants after viral infections. As per the report of Nuruzzaman et al. [36], rice plants upregulated 26 NAC TFs following the pathogen infection to develop resistance against *rice stripe virus* and *rice tungro spherical virus*. Another well-known family of TFs is bZIP, that exists in all eukaryotes. Apart from its regulation of diverse biological processes such as seed development, floral formation, and various biotic and abiotic stresses, it is also an important component of plant defense mechanisms [37]. The bZIP TF involved in plant defense is known as TGA, which interacts with NPR1, and in turn, upregulates the PR genes [38, 39]. This TF is a component of jasmonic acid (JA) and ethylene dependent pathways and the SA-dependent SAR resistance mechanism. Similarly, the bHLH family of TFs is also involved in plant defense. One such known group in bHLH is constituted by the MYC TFs, which are upregulated through the JA signaling pathway. Enhanced MYC levels lead to the production of nicotine in *Nicotiana attenuata*, which in turn increases the plant resistance [40, 41].

Our results indicated that the B-48 genotype showed higher resistance against SCMV, as compared to its wild type counterpart. The higher resistance in B-48 was supported by augmented expression of defense-related transcription factors including WRKY, AP2, bZIP, NAC, and bHLH. The elevated expression level of genes related to the ROS scavenging pathway upregulates the activity of antioxidant enzymes such as SOD, PAL, CAT, and APX, which curtail the levels of ROS radicals, diminishing the oxidative stress in the plant (Additional file 5: Figure S2).

## Conclusions

The transgenic genotype, B-48, was developed for resistance against SCMV. This study evaluated the basis of its resistance against the pathogen and investigated the molecular factors bestowing the essential characteristics for mitigating biotic stress caused by SCMV infection. We observed that the extent of lipid peroxidation was greatly reduced in the B-48 genotype, while its H_2_O_2_ contents also started reducing just after 3 days post-infection. The comparative transcriptomic profiling for ROS producing and antioxidants genes indicated significant differences between B-48 and Badila. The Badila genotype upregulated a higher number of ROS producing unigenes following viral infection. The major transcription factors involved in resistance mechanisms against the pathogens such as WRKY, AP2, NAC, bZIP, and bHLH were found to be highly upregulated in B-48 (PI). The transcriptomic data also agreed with the physiological analysis of the plants. It is suggested that the enhanced defense response allows the B-48 genotype to develop disease resistance and recover from symptoms of SCMV infection, as compared to the wild genotype. This research carries paramount importance in reference to biotic stress tolerance in sugarcane. The study also identifies key players in sugarcane conferring resistance against SCMV and elucidates the defense mechanisms prevailing in this commercially important crop.

## Methods

### Plant samples and SCMV infection

SCMV resistant (B-48, released by Guangxi University) and susceptible (Badila, commercial cultivar) genotypes from the field station of Guangxi University were cultivated in the greenhouse at 24 °C with 16:8 h of light/dark photoperiod. Subsequently, three experimental plants of each genotype at the 4–6 leaf stage were mechanically inoculated with a sap prepared from the crude extract of SCMV infected leaves [42]. Additionally, three experimental non-inoculated plants of both genotypes were used as a control. Non-inoculated Badila and B-48 genotypes, were used as a control plant. Infected (non-transformed) plants produced systemic infections after 14 days. Two leaves from each plant (BI as well as PI) were randomly collected (at 14th day) for further analysis.

### Estimation of ROS (H_2_O_2_) production

H_2_O_2_ contents were measured according to the previously optimized protocol [43]. Frozen leaf samples (approximately 1 g) were mixed with 4 mL of 0.1% (w/v) trichloroacetic acid (TCA). The mixture was then centrifuged at 12,000×g for 15 min. Afterward, the supernatant (1 mL) was mixed with 1 mL of 10 mM potassium phosphate buffer (pH 7.0) and 2 mL of 1 M potassium iodide solution. The H_2_O_2_ concentration was estimated by measuring absorbance at 390 nm. Experiment was repeated three times.

### Quantification of lipid peroxidation

Lipid peroxidation was quantified by estimating the MDA contents, as described previously [44]. In brief, frozen leaf samples were homogenized with 10 mL of 0.25% TBA dissolved in 10% TCA. The obtained extract was heated at 95 °C (30 min) and then chilled on ice. Subsequently, the samples were centrifuged at 5000×g for 15 min. Absorbance was measured at 532 nm via spectrophotometer on three biological replicates.

### Transcriptome analysis

#### RNA extraction

The total RNA from leaves of Badila and B-48 was extracted using Quick-RNA™ Miniprep kit supplied by Zymo Research, USA.

#### Library preparation and RNA sequencing

The sequencing library of each RNA sample was prepared using the NEB Next® Ultra™ RNA Library Prep Kit for Illumina®. Agilent 2100 Bioanalyzer (Agilent Technologies, USA) was used for analyzing each RNA sample, and Illumina paired-end reads were generated employing Illumina HiSeq 2500 platform (Biomarker Technologies, CA, USA). RNA libraries with two replicates of each sample were assembled. For this, the genome-guide approach was utilized employing sugarcane (*S. spontaneum*) as reference [20]. Raw reads quality was preliminarily measured through FastQC (http://www.bioinformatics.babraham.ac.uk/projects/fastqc/), after removing adapters, low quality reads, and poly-N sequences to obtain clean reads, following the procedure adopted in [45].

#### Functional annotation

The search for GO terms and KEGG pathways related to ROS production was conducted using the method described earlier [45].

#### Determination of antioxidant activities

Antioxidant activities were measured according to the previously described protocols. Frozen leaf samples were homogenized in 50 mM potassium phosphate buffer (pH 7.8) and then centrifuged at 10,000×g. Following incubation for 20 min at low temperature (4 °C), the supernatant was utilized to carry out enzyme assays. Each experiment was performed three times.

#### Ascorbate peroxidase (APX) activity

To determine the APX activity, the method of Nakano et al. [46] was adopted. APX activity was calculated by mixing 1 mL of enzyme extract with 50 mM phosphate buffer, 0.5 mM ascorbic acid, 0.1 mM Na_2_-EDTA, and 0.5 mM H_2_O_2_. Absorbance was monitored at 290 nm after supplementing with H_2_O_2_.

#### Catalase (CAT) activity

The CAT activity was measured according to the protocol mentioned by Droillard et al. [47]. Total 3 mL of reaction mixture comprised of 50 mM potassium phosphate buffer (pH 7.0), 10 mM H_2_O_2_, 2 mM Na_2_-EDTA and 0.1 mL of enzyme extract. Absorbance was measured at 240 nm for 1 min.

#### Superoxide dismutase (SOD) activity

The total SOD activity was analyzed according to the method of Droillard et al. [47]. SOD activity was estimated at 560 nm by calculating the inhibition rate of nitroblue tetrazolium (NBT) photochemical activity.

#### Phenylalanine ammonia-lyase (PAL) activity

PAL activity was detected by the estimation of trans-cinnamic acid formation at 290 nm. The reaction mixture (1 mL, pH = 8.8) consisted of enzyme extract, 100 mM Tris-HCl, and 40 mM L-phenylalanine. The mixture was incubated at 37 °C for a period of 30 min. Following the incubation, the reaction was terminated by adding 50 uL of 4 M HCl [48].

#### Real-time quantitative PCR analysis

Total RNA was extracted from the leaf samples using the Tiangen plant RNA extraction kit (Biotech Co., Ltd., Beijing), followed by cDNA synthesis. The qPCR reaction contained 10 μL SYBR Green Master Mix (Roche, Germany), 1 μL of template cDNA, 1 μL of each primer (10 μM), and water up to the final volume of 20 μL. The RNA expression level was normalized to the level of GADPH expression. The amplification was conducted in LightCycler 96 Real-Time PCR system (Roche, China) in three technical replicates. A standard thermal profile for the SYBR master mix was as follows: initial denaturation at 95 °C for 10 min, followed by 40 cycles of denaturation at 95 °C for 15 s, and annealing and extension at 60 °C for 60s. Relative expression levels were calculated with the 2^-ΔΔC^_q_ method, as described earlier [49].

#### Accession numbers

RNA sequencing data used in this article can be found in the Short Read Archive at NCBI (https: //www.ncbi.nlm.nih.gov/sra) using following accession numbers: B-48 (BI) Replicate 1: SRR10058141, B-48 (BI) Replicate 2: SRR10058140, B-48 (PI) Replicate 1: SRR10058139, B-48 (PI) Replicate 2: SRR10058138, Badila (BI) Replicate 1: SRR10058145, Badila (BI) Replicate 2: SRR10058144, Badila (PI) Replicate 1: SRR10058143, Badila (PI) Replicate 2: SRR10058142.

## Supplementary Information

**Additional file 1:** **Figure S1.**Diagrammatic representation of reactive oxygen species (ROS) production and scavenging pathways in various cellular compartments.

**Additional file 2:** **Table S1.**Reads of each genotype (in replicates) with details of mapping to *Sugarcane spontaneum* reference genome.

**Additional file 3:** **Table S2.**List of gene IDs of ROS producing and scavenging enzymes with eggNOG annotation.

**Additional file 4:** **Table S3.**List of primers’ sequences used for qRT-PCR.

**Additional file 5:** **Figure S2.** Schematic representation of the development of resistance in B-48, as compared to Badila. ROS network mediated signaling pathway elucidates the mechanism of SCMV resistance development through regulation of antioxidant pathway and activation of defense associated transcription factors. The red arrow describes the pathway in B-48 while the black arrow represents the mechanism in Badila.

## Data Availability

RNA-sequencing data of this study have been submitted to Short Read Archive at NCBI at https://www.ncbi.nlm.nih.gov/sra/?term=sugarcane+mosaic+virus using following accession numbers: B-48 (BI) Replicate 1: SRR10058141, B-48 (BI) Replicate 2: SRR10058140, B-48 (PI) Replicate 1: SRR10058139, B-48 (PI) Replicate 2: SRR10058138, Badila (BI) Replicate 1: SRR10058145, Badila (BI) Replicate 2: SRR10058144, Badila (PI) Replicate 1: SRR10058143, Badila (PI) Replicate 2: SRR10058142.

## Abbreviations

APX: Ascorbate peroxidase
AsA: Ascorbate
bHLH: Helix loop helix
bZIP: Basic leucine-zipper
CAT: Catalase
DEGs: Differentially expressed genes
DHAR: Dehydroascorbate reductase
FDR: False discovery rate
GPX: Guaiacol peroxidase
GR: Glutathione reductase
GSH: Glutathione
GST: Glutathione s-transferase
MDA: Malondialdehyde
MDAR: Monodehydroascorbate reductase
MDH: Malate dehydrogenase
MDHAR: Monodehydroascorbate reductase
NBT: Nitroblue tetrazolium
PAL: Phenylalanine ammonia-lyase
PCD: Programmed cell death
ROS: Reactive oxygen species
SOD: Superoxide dismutase
TFs: Transcription factors
