# Supplementary figures and images for: Gene expression profiling of reactive oxygen species (ROS) and antioxidant defense system following Sugarcane mosaic virus (SCMV) infection
Source: BMC Plant Biol. 2020 Nov 23;20:532. doi: 10.1186/s12870-020-02737-1 (PMC7685628; doi:10.1186/s12870-020-02737-1)

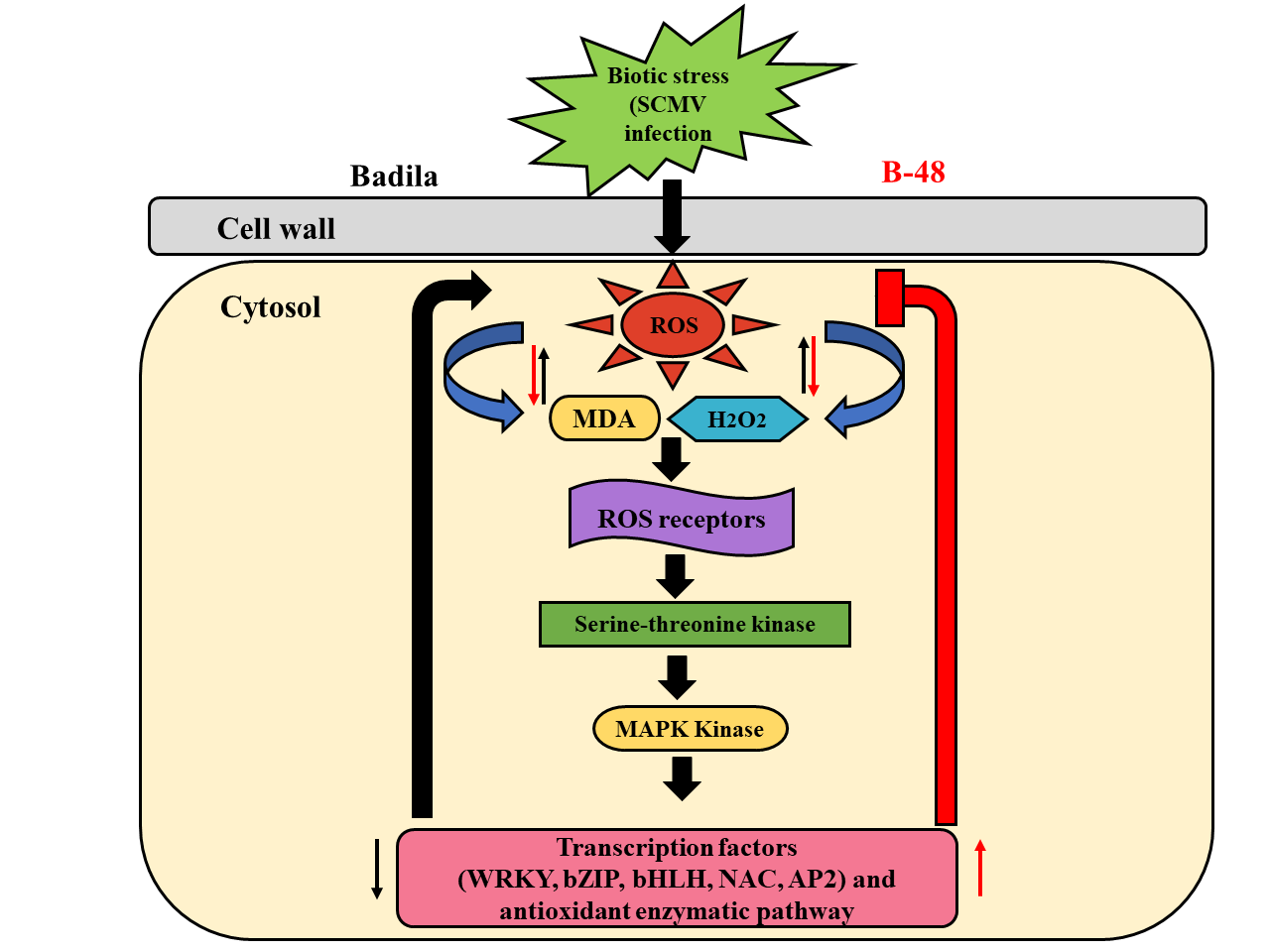

Supplement: Supplementary file 5 — Additional file 5: Figure S2. Schematic representation of the development of resistance in B-48, as compared to Badila. ROS network mediated signaling pathway elucidates the mechanism of SCMV resistance development through regulation of antioxidant pathway and activation of defense associated transcription factors. The red arrow describes the pathway in B-48 while the black arrow represents the mechanism in Badila. [file 12870_2020_2737_MOESM5_ESM.tif]
